# Molecular simulation data for the vapor-liquid phase equilibria of binary mixtures of HFO-1123 with R-32, R-1234yf, R-1234ze(E), R-134a and CO_2_ and their modelling by the PCP-SAFT equation of state

**DOI:** 10.1016/j.dib.2019.104014

**Published:** 2019-05-24

**Authors:** Gabriele Raabe

**Affiliations:** Institut für Thermodynamik, TU, Braunschweig, Germany

**Keywords:** HFO-1123, Refrigerants, VLE, Molecular simulation, PCP-SAFT

## Abstract

In this Data in Brief article, we present predictive data for the vapor-liquid equilibria of the binary mixtures of HFO-1123 with R-32, HFO-1234yf, HFO-1234ze(E), R-134a and CO_2_ from molecular simulation. The VLE in the binary mixtures are then modeled by the PCP-SAFT equation of state. Therefore we determined PCP-SAFT parameters for the pure HFO compounds as well as binary interaction parameters for all mixtures. The simulation data and the PCP-SAFT modelling are discussed in a related research article (Raabe, 2019).

**Table:** Specifications Table

Subject area	Chemistry, chemical engineering
More specific subject area	Molecular simulation, thermophysical properties
Type of data	Tables, figure
How data was acquired	The VLE data was acquired by Gibbs Ensemble Monte Carlo (GEMC) simulation using the code TOWHEE [2] and employing force field models from previous work [3], [4], [5]. The simulations were performed on the HPC compute server Phoenix of the TU Braunschweig
Data format	Filtered, analyzed.
Experimental factors	Simulations were performed in NPT ensemble. Each system consisted of 432 molecules. For each data point, the simulation was equilibrated for 200,000 cycles. The production runs consisted of 300,000–500.000 cycles. Standard deviations of all ensemble averages were calculated by the standard block averaging technique.
Experimental features	GEMC molecular simulation results for the compositions and saturated densities of the VLE in the binary mixtures for imposed temperatures and pressures. The data are in the temperature range T = (220–310) K
Data source location	Braunschweig, Germany, TU Braunschweig, Institut für Thermodynamik
Data accessibility	data is with this article
Related research article	Molecular Simulation Studies on Refrigerants. Past – Present – Future. https://doi.org/10.1016/j.fluid.2018.12.022[1]

**Table:** 

**Value of the Data** • This article provides first data for the VLE properties of binary mixtures of HFO-1123 with R-32, R-1234yf, R-1234ze(E), R-134a and CO_2_, which are discussed as new refrigerants blends • The new PCP-SAFT parameters (EOS parameters ε, σ, μ and Q) provided in this article allow for an accurate modelling of the pure compounds HFO-1123, -1234yf,-1234ze(E) • The k_ij_ interaction parameters in this article allow for a computation of the VLE properties of binary mixtures of HFO-1123 with R-1234yf, R-1234ze(E), R-32, R-134a, CO_2_ by the PCP-SAFT equation of state • The data and modelling approach provided in this article may be used by researchers in the field of refrigeration to evaluate the performance of new HFO-1123 based refrigerant blends

## 1 Data

### 1.1 Molecular simulation data

These data comprise GEMC simulation data for the compositions of the liquid and vapor phase, and for the saturation densities of the vapor-liquid equilibria of the mixtures of HFO-1123 with R-1234yf, R-1234ze(E), R-32, R-134a, CO_2_ (see Table 1, Table 2).

**Table 1:** Data from GEMC simulations for the VLE in binary mixtures of HFO-1123 with R-32, R-1234yf, R-1234ze(E) and R-134a: mole fraction in the saturated liquid (x) and vapor phase (y), and saturated densities ρ^L^ and ρ^V^. Values in parentheses denote standard deviations.

T (K)	p(MPa)	x_1123_ (mol mol^−1^)	y_1123_ (mol mol^−1^)	ρ^L^ (kg m^−3^)	ρ^V^ (kg m^−3^)
HFO-1123 + R-32					
220.0	0.10	0.082 (0.003)	0.167 (0.013)	1254.0 (3.2)	3.3 (0.1)
	0.11	0.216 (0.014)	0.351 (0.006)	1270.0 (1.6)	3.9 (0.1)
	0.12	0.361 (0.013)	0.500 (0.011)	1283.5 (1.5)	4.6 (0.1)
	0.13	0.593 (0.002)	0.671 (0.003)	1304.7 (1.0)	5.3 (0.1)
230.0	0.17	0.134 (0.009)	0.235 (0.012)	1229.2 (1.1)	5.5 (0.1)
	0.18	0.216 (0.013)	0.338 (0.014)	1238.5 (1.4)	6.2 (0.1)
	0.19	0.321 (0.009)	0.446 (0.009)	1248.5 (1.3)	6.9 (0.1)
	0.20	0.414 (0.008)	0.530 (0.005)	1258.0 (2.8)	7.5 (0.1)
	0.21	0.594 (0.008)	0.645 (0.014)	1260.4 (9.2)	8.1 (0.2)
270.0	0.80	0.117 (0.004)	0.174 (0.006)	1087.8 (0.7)	24.3 (0.1)
	0.85	0.242 (0.008)	0.319 (0.008)	1098.5 (1.4)	27.7 (0.1)
	0.90	0.385 (0.004)	0.456 (0.004)	1110.3 (1.2)	31.3 (0.1)
290.0	1.45	0.116 (0.001)	0.157 (0.002)	998.7 (2.2)	44.5 (0.2)
	1.50	0.173 (0.001)	0.225 (0.002)	1005.9 (1.9)	47.7 (0.3)
	1.55	0.268 (0.002)	0.324 (0.001)	1010.3 (0.5)	51.7 (0.1)
	1.60	0.350 (0.005)	0.404 (0.004)	1016.9 (1.6)	55.6 (0.3)
HFO-1123 + R-1234yf					
220.0	0.05	0.201 (0.008)	0.558 (0.012)	1339.4 (3.1)	2.7 (0.1)
	0.075	0.443 (0.016)	0.800 (0.012)	1335.6 (2.3)	3.7 (0.1)
	0.1	0.657 (0.024)	0.905 (0.008)	1334.0 (2.7)	4.8 (0.1)
240.0	0.15	0.302 (0.015)	0.638 (0.019)	1277.0 (2.5)	7.4 (0.1)
	0.2	0.459 (0.012)	0.771 (0.013)	1275.8 (3.0)	9.5 (0.1)
	0.3	0.863 (0.009)	0.960 (0.003)	1266.6 (2.2)	13.5 (0.1)
270.0	0.4	0.183 (0.016)	0.402 (0.025)	1185.3 (2.6)	20.0 (0.2)
	0.5	0.319 (0.010)	0.581 (0.011)	1182.2 (1.3)	23.8 (0.1)
	0.65	0.554 (0.013)	0.782 (0.009)	1171.1 (2.0)	29.4 0.1)
	0.75	0.695 (0.006)	0.866 (0.003)	1165.4 (1.7)	33.4 (0.1)
	0.85	0.830 (0.010)	0.932 (0.005)	1158.1 (3.1)	37.3 (0.1)
290.0	0.75	0.202 (0.009)	0.396 (0.011)	1111.2 (3.3)	37.1 (0.2)
	1.0	0.408 (0.010)	0.630 (0.011)	1101.7 (2.1)	47.0 (0.3)
	1.25	0.628 (0.007)	0.804 (0.005)	1087.4 (2.5)	56.6 (0.2)
	1.5	0.836 (0.008)	0.924 (0.004)	1069.6 (2.9)	67.2 (0.2)
310.0	1.1	0.087 (0.008)	0.173 (0.002)	1035.2 (1.9)	59.0 (0.4)
	1.25	0.183 (0.009)	0.326 (0.012)	1030.0 (3.0)	64.5 (0.4)
	1.5	0.327 (0.010)	0.508 (0.012)	1020.2 (2.3)	74.7 (0.4)
	1.75	0.474 (0.015)	0.652 (0.015)	1004.7 (4.6)	84.6 (1.0)
	2.0	0.602 (0.008)	0.755 (0.006)	992.6 (2.5)	95.9 (0.5)
	2.25	0.747 (0.006)	0.854 (0.004)	969.8 (2.2)	106.4 (0.7)
HFO-1123 + R-1234ze(E)					
250.0	0.2	0.318 (0.009)	0.726 (0.007)	1306.6 (2.0)	9.2 (0.1)
	0.3	0.527 (0.008)	0.863 (0.007)	1288.5 (0.6)	13.4 (0.1)
	0.4	0.786 (0.022)	0.952 (0.007)	1257.8 (4.7)	17.5 (0.1)
270.0	0.3	0.148 (0.005)	0.435 (0.013)	1260.1 (1.9)	14.4 (0.1)
	0.4	0.282 (0.007)	0.631 (0.012)	1249.2 (1.5)	18.2 (0.1)
	0.6	0.550 (0.020)	0.839 (0.011)	1217.3 (4.4)	26.0 (0.1)
	0.8	0.802 (0.017)	0.942 (0.006)	1182.8 (5.4)	34.8 (0.3)
290.0	0.6	0.172 (0.005)	0.420 (0.005)	1193.4 (2.8)	28.3 (0.1)
	0.8	0.355 (0.006)	0.653 (0.010)	1167.2 (1.6)	35.6 (0.2)
	1.0	0.486 (0.016)	0.759 (0.010)	1153.3 (6.1)	44.1 (0.3)
	1.2	0.643 (0.014)	0.853 (0.010)	1128.4 (4.3)	52.5 (0.1)
	1.4	0.791 (0.007)	0.921 (0.003)	1101.5 (3.4)	61.5 (0.1)
310.0	0.8	0.049 (0.004)	0.131 (0.007)	1129.4 (3.2)	40.3 (0.2)
	1.0	0.157 (0.006)	0.346 (0.013)	1117.1 (2.5)	47.9 (0.2)
	1.2	0.266 (0.013)	0.500 (0.019)	1101.0 (5.6)	55.9 (0.5)
	1.6	0.458 (0.005)	0.690 (0.007)	1074.1 (1.8)	73.4 (0.7)
	2.0	0.652 (0.012)	0.824 (0.007)	1036.1 (4.5)	91.6 (0.3)
	2.4	0.832 (0.008)	0.922 (0.004)	989.7 (4.3)	112.3 (0.5)
HFO-1123 + R-134a					
250.0	0.2	0.203 (0.014)	0.485 (0.022)	1335.3 (2.8)	9.4 (0.1)
	0.3	0.476 (0.017)	0.763 (0.011)	1303.0 (3.4)	13.4 (0.1)
	0.4	0.728 (0.017)	0.901 (0.010)	1270.1 (3.4)	17.7 (0.1)
270.0	0.4	0.172 (0.010)	0.389 (0.018)	1274.7 (2.3)	18.4 (0.1)
	0.5	0.337 (0.008)	0.603 (0.011)	1252.7 (1.8)	22.2 (0.1)
	0.6	0.489 (0.008)	0.737 (0.005)	1230.6 (1.8)	26.2 (0.1)
	0.7	0.608 (0.017)	0.817 (0.010)	1215.1 (4.9)	30.5 (0.1)
	0.825	0.780 (0.014)	0.908 (0.006)	1188.7 (3.6)	36.0 (0.1)
290.0	0.8	0.206 (0.006)	0.399 (0.003)	1199.1 (0.7)	36.4 (0.1)
	1.0	0.400 (0.012)	0.624 (0.012)	1169.4 (3.3)	44.3 (0.2)
	1.2	0.565 (0.013)	0.758 (0.009)	1142.0 (4.9)	52.8 (0.3)
	1.4	0.752 (0.018)	0.880 (0.019)	1109.0 (4.4)	61.7 (0.2)
310.0	1.2	0.126 (0.007)	0.243 (0.013)	1128.3 (1.5)	56.1 (0.1)
	1.4	0.232 (0.006)	0.396 (0.010)	1112.6 (2.5)	64.6 (0.4)
	2.2	0.694 (0.011)	0.816 (0.008)	1019.2 (4.4)	101.9 (0.5)
	2.4	0.794 (0.006)	0.880 (0.004)	994.9 (2.8)	111.8 (0.2)

**Table 2:** Data from GEMC simulations for the VLE in binary mixture CO_2_ + HFO-1123: mole fraction in the saturated liquid (x) and vapor phase (y), and saturated densities ρ^L^ and ρ^V^. Values in parentheses denote standard deviations.

T (K)	p(MPa)	x_CO2_ (mol mol^−1^)	y_CO2_ (mol mol^−1^)	ρ^L^ (kg m^−3^)	ρ^V^ (kg m^−3^)
CO_2_ + HFO-1123					
250.0	1.0	0.382 (0.005)	0.654 (0.006)	1171.2 (2.2)	30.6 (0.5)
	1.5	0.811 (0.011)	0.921 (0.006)	1087.4 (3.6)	40.0 (0.2)
270.0	1.5	0.252 (0.006)	0.467 (0.010)	1109.7 (2.6)	51.8 (0.6)
	2.0	0.475 (0.005)	0.692 (0.006)	1069.4 (3.2)	61.1 (0.7)
	2.2	0.570 (0.007)	0.763 (0.006)	1051.0 (2.4)	65.1 (0.7)
	2.5	0.700 (0.004)	0.845 (0.004)	1022.7 (3.4)	70.6 (1.3)
	2.7	0.794 (0.007)	0.897 (0.005)	1001.8 (3.4)	74.5 (1.0)
290.0	2.5	0.237 (0.005)	0.401 (0.008)	1015.9 (4.6)	93.3 (1.5)
	3.0	0.378 (0.003)	0.561 (0.006)	984.7 (4.8)	102.0 (2.6)
	3.5	0.520 (0.005)	0.683 (0.005)	958.0 (3.5)	115.3 (1.3)
	4.0	0.651 (0.005)	0.783 (0.004)	923.6 (4.1)	125.2 (2.2)
	4.5	0.784 (0.007)	0.870 (0.005)	889.3 (6.0)	137.4 (3.5)

### 1.2 PCP-SAFT parameters

**Fig. 1:**
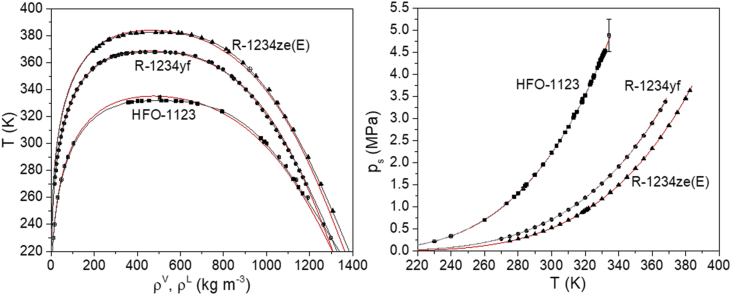
Correlation of the VLCC and vapor pressure curve of R-1234ze(E)=▲, R-1234yf=● and HFO-1123=■by the PCP-SAFT (red line) and available EOS models in REFPROP [8](dark grey line). Shown as filled symbols are experimental VLE data for R-1234ze(E) ([7], [9], [10], [11]), R-1234yf ([12]) and HFO-1123 ([13], [14]). Also given are GEMC simulation results ⊠ for HFO-1123 that were employed in the fitting (∃, [5]).

We here present PCP-SAFT parameters for the pure compounds HFO-1123, -1234yf, and -1234ze(E). Fig. 1 shows a comparison of the correlation of the VLCC and vapor pressure curve of R-1234ze(E), R-1234yf and HFO-1123 by the PCP-SAFT (red line) and available EOS models in REFPROP (dark grey line) as well as with experimental data. For the correlation of the mixture data, we employed PCP-SAFT parameters from literature for the pure compounds R-32, R-134a and CO_2._ The parameters are also give in Table 3.

**Table 3:** PCP-SAFT parameters for the refrigerants studied in this work.

Refrigerant	*M* g mol^−1^	*m*	σ* A	ε*/k K	μ* D	Q DÅ	Source
HFO-1123	82.02	1.9620	3.45868	172.948		5.348	this work
R-32	52.02	2.47192	2.79714	161.6614	1.978		Vinš et al. [6]
R-134a	102.03	3.14704	3.04554	165.3555	2.058		Vinš et al. [6]
R-1234yf	114.04	1.63761	4.23860	185.2610	0.9887	10.35	this work
R-1234ze(E)	114.04	1.97291	3.918	188.7388		8.98	this work
CO_2_	44.01	1.5131	3.1869	163.33		4.4	Gross [7]

We also provide the fitted interaction parameters k_ij_ for the EOS for the binary mixtures HFO-1123 with R-1234yf, R-1234ze(E), R-32, R-134a, CO_2._ The k_ij_ along with the relative average deviations (RAD %) of the pressure and saturated densities, and absolute average deviations (AAD) of the molar vapor composition of the correlations from the GEMC simulation results are summarized in Table 4. In [1] we provide depictions of calculated isotherms of all mixtures in comparison with the simulation data and calculations using REFPROP.

**Table 4:** PCP-SAFT Interaction parameters *k*_*ij*_, and resulting deviation from the GEMC simulation.

Refrigerant mixture	*k*_*ij*_	Δ*p, RAD* %	Δ*y, AAD* mol mol^−1^	Δ*ρ*^*L*^*, RAD* %	Δ*ρ*^*V*^*, RAD* %
HFO-1123 + R-32	0.0358	1.5	0.0209	1.3	5.6
HFO-1123 + R-1234ze(E)	0.0048	1.6	0.0053	2.2	1.7
HFO-1123 + R-1234yf	−0.0083	2.6	0.0084	1.0	3.4
HFO-1123 + R-134a	−0.0003	1.2	0.0033	0.6	1.9
CO_2_ + HFO-1123	0.0030	1.2	0.0039	1.7	3.3

## 2 Methods

### 2.1 Molecular simulation

Predictions for the vapor-liquid equilibria of the binary mixtures were derived by Monte Carlo Gibbs ensemble (GEMC, [15]) simulations in the NPT ensemble using the simulation code TOWHEE [2]. Each system consisted of 432 molecules in total, but depending on the mixture studied, the number of the molecules of both components were varied to yield a feed composition within the two phase region. The Ewald sum technique [16] was employed to deal with the electrostatic interactions with a cut-off radius adjusted to half the box length, whereas the cut-off radius for the Lennard-Jones interactions was set to 12 Å. Standard long-range corrections to the energy and pressure were applied (e.g. Ref. [17]). For each data point, the simulation was equilibrated for 200,000 cycles. The production runs consisted of 300,000–500.000 cycles from which ensemble averages for the compositions and saturated densities of the coexisting phases for the imposed temperature and pressure were determined. Standard deviations of all ensemble averages were calculated by the standard block averaging technique (e.g. Refs. [17], [18]).

### 2.2 PCP-SAFT-modelling

For CO_2_, we used the PCP-SAFT parameters proposed by Gross [7] whereas the parameters of R-32 and R-134a were taken from Vinš et al. [6]. In this work, we derived PCP-SAFT parameters for the compounds HFO-1123, R-1234yf and R-1234ze(E). For the tetraflouropropenes R-1234yf and R-1234ze(E), the PCP-SAFT parameters were determined by fitting calculated vapor pressure and liquid densities to experimental data, and we therefore employed the same experimental data set as in the fitting of the PC-SAFT model in our previous work [4]. As experimental data for HFO-1123 in literature are limited [13], [14], we also employed our molecular simulation results [5] in the fitting of the EOS parameters for this compound.

To model the refrigerant mixtures, common combining rules for the PCP-SAFT parameters are used

$$\varepsilon_{ij}^{*}=\sqrt{\varepsilon_{ii}^{*}\varepsilon_{jj}^{*}\;}\left(1-k_{ij}\right)\;,\sigma_{ij}^{*}=\frac{\sigma_{ii}^{*}+\sigma_{jj}^{*}}{2}$$

that employ an interaction parameter k_ij_ for the interaction energy $\varepsilon_{ij}^{*}$ between unlike segments. The interaction parameters k_ij_ were derived by fitting to the GEMC simulation results for the VLE of the binary mixtures presented in this work.
