# Supplementary material for: Molecular simulation data for the vapor-liquid phase equilibria of binary mixtures of HFO-1123 with R-32, R-1234yf, R-1234ze(E), R-134a and CO2 and their modelling by the PCP-SAFT equation of state
Source: Data Brief. 2019 May 24;25:104014. doi: 10.1016/j.dib.2019.104014 (PMC6661389; doi:10.1016/j.dib.2019.104014)
Supplement: Multimedia component 1 [file mmc1.pdf]

AUTHOR DECLARATION

I wish to confirm that there are no known conflicts of interest associated with this publication and there has been no significant financial support for this work that could have influenced its outcome.

I confirm that the manuscript has been read and approved by all named authors and that there are no other persons who satisfied the criteria for authorship but are not listed.

I confirm that we have given due consideration to the protection of intellectual property associated with this work and that there are no impediments to publication, including the timing of publication, with respect to intellectual property. In so doing I confirm that we have followed the regulations of our institutions concerning intellectual property.

We understand that the Corresponding Author is the sole contact for the Editorial process (including Editorial Manager and direct communications with the office). He/she is responsible for communicating with the other authors about progress, submissions of revisions and final approval of proofs. We confirm that we have provided a current, correct email address which is accessible by the Corresponding Author and which has been configured to accept email from

G.Raabe@tu-bs.de

Gabriele Raabe, May 6th 2019
